# Pervasive Mitochondrial Sequence Heteroplasmy in Natural Populations of Wild Carrot, *Daucus carota* spp. *carota* L

**DOI:** 10.1371/journal.pone.0136303

**Published:** 2015-08-21

**Authors:** Jennifer R. Mandel, David E. McCauley

**Affiliations:** 1 Department of Biological Sciences, The University of Memphis, 3700 Walker Avenue, Memphis, Tennessee, United States of America; 2 W. Harry Feinstone Center for Genomic Research, The University of Memphis, 3774 Walker Avenue, Memphis, Tennessee, United States of America; 3 Department of Biological Sciences, Vanderbilt University, VU Station B, Box 351634, Nashville, Tennessee, United States of America; University of the Sunshine Coast, AUSTRALIA

## Abstract

Exceptions to the generally accepted rules that plant mitochondrial genomes are strictly maternally inherited and that within-individual sequence diversity in those genomes, i.e., heteroplasmy, should be minimal are becoming increasingly apparent especially with regard to sequence-level heteroplasmy. These findings raise questions about the potential significance of such heteroplasmy for plant mitochondrial genome evolution. Still studies quantifying the amount and consequences of sequence heteroplasmy in natural populations are rare. In this study, we report pervasive sequence heteroplasmy in natural populations of wild carrot, a close relative of the cultivated crop. In order to assay directly for this heteroplasmy, we implemented a quantitative PCR assay that can detect and quantify intra-individual SNP variation in two mitochondrial genes (*Cox1* and *Atp9*). We found heteroplasmy in > 60% of all wild carrot populations surveyed and in > 30% of the 140 component individuals that were genotyped. Heteroplasmy ranged from a very small proportion of the total genotype (e.g., 0.995:0.005) to near even mixtures (e.g., 0.590:0.410) in some individuals. These results have important implications for the role of intra-genomic recombination in the generation of plant mitochondrial genome genotypic novelty. The consequences of such recombination are evident in the results of this study through analysis of the degree of linkage disequilibrium (LD) between the SNP sites at the two genes studied.

## Introduction

Mitochondrial heteroplasmy is the co-occurrence of two or more divergent mitochondrial genotypes within an individual, and for the purpose of this study, we consider heteroplasmy at the level of DNA sequence variation, often single nucleotide polymorphisms (SNPs), for one or more mitochondrial genes recognizing that the broader definition can include structural variation and substoichiometric molecules thoroughly discussed in a nice review by Woloszynska [1]. Plant mitochondrial genomes are usually transmitted maternally; and as such, this strict uni-parental inheritance should result in sequence homoplasmy, or within-individual genetic homogeneity, by preventing the mixing of differing mitochondrial genomes at fertilization. However, as McCauley [2] points out in a recent *Tansley Review*, it is becoming increasingly apparent that bi-parental inheritance via occasional paternal transmission (leakage) of plant mitochondrial genomes can generate heteroplasmy and thus within-individual mitochondrial genome diversity. The occurrence of such heteroplasmy can enhance the possibility that inter-molecular recombination between divergent genetic partners will result in novel multi-locus genotypic combinations of potential significance for plant mitochondrial genome evolution. Moreover, Burt and Trivers [3] have suggested that in plant species exhibiting gynodioecy (a mating system consisting of a mixture of females and hermaphrodites) and cytoplasmic male sterility (CMS; [4]), selection could favor paternal leakage of mitochondrial genomes. Given that gynodioecy is the second most common mode of reproduction in angiosperms [5], the potential significance for the evolution of the mating system and the plant mitochondrial genome is quite intriguing.

Data from the gynodioecious species *Silene vulgaris* (Family: Caryophyllaceae), the bladder campion, have provided considerable information regarding mitochondrial heteroplasmy, paternal leakage, and recombination [2]. Studies from natural populations first demonstrated evidence for both mitochondrial heteroplasmy and inter-molecular recombination and provided indirect support for paternal leakage of mitochondrial genomes [6–8]. Following these studies, direct evidence for the paternal inheritance of mitochondrial genomes in *S*. *vulgaris* was demonstrated from formal crosses [9]. These studies of *S*. *vulgaris* utilized quantitative real time PCR (qPCR) both to detect and to quantify mitochondrial gene SNPs used to define heteroplasmy. While such heteroplasmy was found in > 10% of *S*. *vulgaris* individuals assayed from natural populations by Pearl *et al*. [8], it remains largely unknown how widely distributed mitochondrial genome heteroplasmy might be across diverse plant taxa, especially in gynodioecious species, and therefore the broad significance of mitochondrial heteroplasmy for plant mitochondrial genome dynamics is largely unknown.

*Daucus carota* ssp. *carota* (Family: Apiaceae), a.k.a. Queen Anne’s Lace or wild carrot, is the widespread, weedy progenitor of cultivated carrot, *D*. *c*. ssp. *sativus*, and the two subspecies are inter-fertile and can readily cross-pollinate with one another [10]. Many populations of wild carrot exhibit gynodioecy [11] and have recently served as a genetic resource for breeders of domesticated carrot in that some contain cytoplasmic male sterility (CMS) elements (those that render hermaphrodites functionally female) useful in certain breeding strategies [12]. Indeed, a statistical association is known between certain mitochondrial gene markers and CMS [12]. In view of this, Mandel *et al*. [13] conducted population genetic studies of natural populations of *D*. *c*. ssp. *carota* in order to characterize the frequency and geographic distribution of the various mitochondrial SNP and indel marker variants of interest, with special reference to a SNP in the stop codon of the mitochondrial gene *Atp9* that serves as one of the CMS markers[14]. One observation made in that study was that the diversity of multi-locus mitochondrial genotypes implied products of inter-molecular recombination in the presence of mitochondrial genome heteroplasmy (because, barring mutation, heteroplasmy is required for recombination to create novel genotypes) [13]. The conclusion of the presence of heteroplasmy was speculative in that no direct evidence for it was observed. More recently, Szklarczyk *et al*. [15] have demonstrated the presence of *Atp9* heteroplasmy in two cultivated petaloid, CMS carrot lines and their respective fertile maintainer lines. These results suggest the possibility that natural populations of *D*. *c*. ssp. *carota* could harbor levels of mitochondrial genome heteroplasmy (possibly as a consequence of paternal leakage) sufficient to influence the evolution of that genome and its utility as a genetic resource for carrot breeders.

Here we utilize qPCR genotyping of SNP markers found in two mitochondrial genes present in wild carrot to assay for heteroplasmy within 140 individuals sampled from thirteen open-pollinated natural populations located in the eastern United States. We find that heteroplasmy across these populations is quite pervasive. We discuss the implications of these findings including the potential role of intra-genomic recombination in generating mitochondrial genotypic novelty and for the proposed relationship between the presence of CMS-based gynodioecy and paternal mitochondrial leakage.

## Materials and Methods

### Sampling and assay strategy

For this study, either leaf material or seeds (subsequently grown in a greenhouse to obtain leaf material) were collected from 140 wild *D*. *carota* ssp. *carota* L. (hereafter simply *D*. *carota*) individuals found in one of thirteen sites located in the eastern United States (Table 1). All leaves and seeds were collected from publicly available land, and no specific permissions were required for these locations. Moreover, this study did not involve endangered or protected species. Genomic DNA was isolated from a 2 cm square portion of leaf tissue obtained from individual leaves using the DNeasy Plant Mini Kit and associated protocols (Qiagen, Valencia, CA, USA). Our previous work demonstrated substantial within and among population genetic allelic variation in several mitochondrial genes [13]. For the present study, we used known single nucleotide polymorphisms (SNPs) in the coding sequences of two of these genes (*Cox1* and *Atp9*) as markers to investigate the potential for mitochondrial heteroplasmy, using a genotyping strategy modeled on the methodologies of prior studies in *S*. *vulgaris* [6,8,9]. The *Cox1* assay distinguishes a T/C polymorphism in the coding region of the gene, and the *Atp9* assay distinguishes a T/C polymorphism found at the first nucleotide position of the *Atp9* stop codon [12,14]. Mandel *et al*. [13], found a third *Atp9* SNP variant (an ‘A’) at this site in natural populations that was not known previously from cultivated carrot lines. Note that our current *Atp9* assay will therefore only detect T/C heteroplasmy in our samples and underestimate heteroplasmy when the ‘A’ is involved.

**Table 1:** Collection sites of wild carrot, *Daucus carota* ssp. *carota*, populations.

Population Name	State	County
Bartlett	Massachusetts	Nantucket
Moores	Massachusetts	Nantucket
New Street	Massachusetts	Nantucket
Polpis	Massachusetts	Nantucket
Ocean City	New Jersey	Cape May
Beaver	New York	Delaware
Bowl	New York	Delaware
Cemetery	New York	Delaware
Grand Gorge	New York	Delaware
Gravel/Railroad	New York	Schoharie
UT	New York	Delaware
Bean Pot	Tennessee	Cumberland
Bett’s	Tennessee	Davidson

### Quantitative PCR assay

We designed two custom TaqMan SNP genotyping assays (Applied Biosystems, Foster City, CA, USA)–one for the *Cox1* gene and one for the *Atp9* gene. For each assay, two allele-specific TaqMan probes containing distinct fluorescent dyes and a PCR forward and reverse primer pair were designed in order to detect the specific SNP targets. Each probe included a 5’ reporter dye (VIC or 6-FAM), a 3’ non-fluorescent quencher, and a minor groove binder moiety attached to the quencher molecule. These custom assays can be ordered from ABI by providing assay numbers AHBKC6A for *Cox1* and AH5I5BL for *Atp9* (see Table A in S1 File for primer and probe sequences included in the assays). QPCR reactions were performed using 12.5 μl of TaqMan Genotyping Master Mix (Applied Biosystems), 0.6 μl of QPCR assay (*Atp9* or *Cox1*), 9.9 μl dH_2_O, and 2 μl of DNA ([10 ng/μl]). QPCR cycling was as follows: stage 1–50°C, 2:00 min; stage 2–95°C, 10:00 min; stage 3–40 reps, 95°C, 0:15 min followed by 60°C, 1:00 min. All reactions were conducted using an ABI 7300 real-time PCR system. Note that the ‘reciprocal knockback’ method [8], designed to make very low frequency minority SNP variants within heteroplasmic individuals more detectable, was not employed here owing to a lack of unique restriction sites within the *Cox1* and *Atp9* probe sequences.

The QPCR experiment yields a Ct, or cycle threshold, value for each probe. The Ct value is inversely proportional to the number of target copies in the original sample. When two probes are used in an assay as above, the difference between the probe-specific Ct values is a function of the relative copy number of their respective targets. In a homoplasmic individual, one of the probes will not produce sufficient fluorescence to generate a Ct value (a result designated here as “U” or undetermined). In the assays reported here, apparently homoplasmic individuals displayed Ct values of approximately 21 if homoplasmic for one of the two *Cox1* variants and 26 if homoplasmic for one of the two *Atp9* variants (and U for the other variant). In an individual heteroplasmic for the two alleles/haplotypes that are detectable by a given pair of probes, the Ct value for the more common, or primary, allele/haplotype should be the smaller of the two.

For this study, each gene was assayed twice for every individual. For each gene, the only individuals included in the final data set were those, which displayed a Ct value other than “U” (i.e. detectable Ct < 40) for at least one of the two SNP variants for both replicate assays of that gene. This resulted in a data set containing 140 individual *Cox1* genotypes, of which 50 individuals were also genotyped for *Atp9*. It is assumed that in some individuals, unknown SNP variants within the sequence targeted by the *Cox1* or *Atp9* probes could render the genotype of that individual undetectable by the current QPCR assays if homoplasmic for that variant. Further, it is known that in natural populations of *D*. *carota* > 25% of individuals carry neither the ‘T’ nor the ‘C’ *Atp9* SNP genotype, but rather harbor an ‘A’ nucleotide at the T/C SNP site targeted by the *Atp9* assay [13]. Such ‘A’ individuals could not be genotyped by the current T/C *Atp9* assay.

### Artificial heteroplasmy mixture experiment

**Fig 1:**
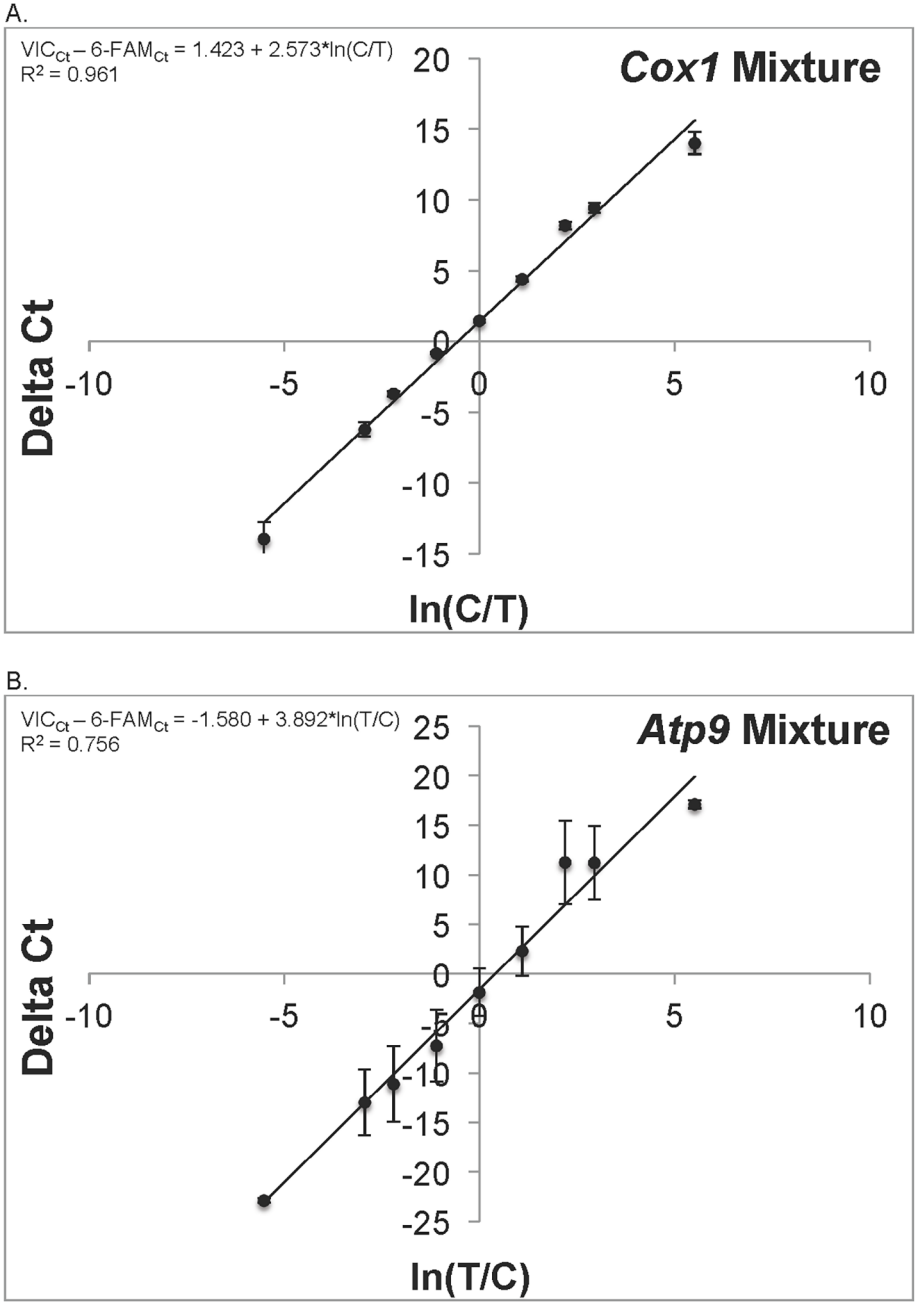
QPCR Mixture Experiments. A) The mean difference in probe-specific Ct values as a function of known relative concentrations of *Cox1* haplotypes. B) The mean difference in probe-specific Ct values as a function of known relative concentrations of *Atp9* haplotypes. Standard error bars for Ct value differences are also presented, based on six replicates for each relative concentration, along with the line derived from linear regression of mean difference in Ct values on log known relative concentration. Linear regression equations derived from these results are shown on the figure and are used to estimate relative copy number from observed Ct values for each probe pair in field-sampled adults.

In order to establish the relationship between differences in Ct value and relative copy numbers for the *Cox1* and *Atp9* combinations, the following artificial heteroplasmy mixture experiment was performed following the strategy employed previously by Pearl *et al*. [8]. Based on an initial assay, we chose individuals that showed only one or the other allele/haplotype in at least three QPCR reactions for an artificial heteroplasmy mixture experiment. For each gene-specific assay, we chose three pairs of two individuals that showed only evidence of a T or a C SNP for that gene. We combined DNA of these individuals to generate the following T vs. C percent mixtures respectively: 100/0, 95/5 90/10, 75/25, 50/50, 25/75, 10/90, 5/95, 0/100 that mimicked various possible levels of heteroplasmy. These mixtures were replicated six times. Following the methods of Pearl *et al*. [8], we analyzed the resulting Ct data in a linear regression framework (Fig 1). For each assay, the difference between the probe-specific Ct values should be a function of the known relative concentrations of the two target SNPs. The log transformation of the known initial relative concentrations is expected to be linear relative to the difference in Ct values because PCR initially results in exponential growth in amplicon number [8]. This linear function was established by linear regression using JMP 11.1.1 (SAS Institute Inc.) and data from the mixture experiment. The resulting equation was then rearranged algebraically and used to estimate the unknown relative copy number (i.e., the heteroplasmy score) within an individual from the difference in Ct values between the two probes found in an assay. In these calculations, a score of 60 was arbitrarily assigned to those Ct values of ‘U’ that would allow a numerical solution that would not indicate the presence of an undetected SNP variant.

### Sequencing validation

To confirm the accuracy of the genotyping obtained from our QPCR assays, we performed PCR amplification and direct Sanger Sequencing in twelve samples (italicized in Table 2) sequenced separately for both *Cox1* and *Atp9*. PCR primers and reaction conditions were as in Mandel *et al*. [13]. DNA sequencing was performed by the Molecular Resource Core Facility at the University of Tennessee Health Science Center employing Applied Biosystems Big Dye v3.1 Reaction Mix at 1/10X strength combined with an appropriate nanogram quantity of PCR product and 10 pmol of the relevant primer in a total volume of 12 μl; all PCR protocols and template/primer ratios were those suggested by the manufacturer. The Applied Biosystems BDXterminator system was used to remove salts, primers and unincorporated nucleotides from the labeling reaction; samples were analyzed using an Applied Biosystems 3130XL Genetic Analyzer. Sequences were viewed and aligned using the Sequencher Software (v5, Gene Codes, Ann Arbor, MI), and sequences were aligned with the aid of published *D*. *carota* sequences [12,14].

**Table 2:** Heteroplasmy scores and SNP genotypes for 140 *Cox1* and 50 *Atp9* individuals as given by relative proportion of the T SNP for both genes.

Population	*Cox1*	Geno.	*Atp9*	Geno.	Population	*Cox1*	Geno.	*Atp9*	Geno.
Bartlett	1.000	T	---	---	Gravel/Railroad	0.000	C	**0.945**	T/C
Bartlett	1.000	T	---	---	Gravel/Railroad	0.000	C	1.000	T
Bean Pot	0.000	C	1.000	T	*Gravel/Railroad*	**0.035**	C/T	1.000	T
*Beaver*	0.000	C	**0.934**	T/C	Gravel/Railroad	**0.031**	C/T	0.996	T
Beaver*	**0.007**	C/T	**0.980**	T/C	Gravel/Railroad	0.000	C	---	---
Beaver	**0.012**	C/T	0.996	T	Gravel/Railroad	0.000	C	---	---
Beaver	1.000	T	0.998	T	Gravel/Railroad	0.001	C	---	---
*Beaver*	**0.012**	C/T	0.998	T	Gravel/Railroad	0.002	C	---	---
Beaver	**0.008**	C/T	0.998	T	Gravel/Railroad	0.004	C	---	---
Beaver	0.000	C	0.999	T	*Gravel/Railroad*	0.004	C	---	---
Beaver	0.000	C	1.000	T	Gravel/Railroad	0.004	C	---	---
Beaver	0.000	C	---	---	Gravel/Railroad	**0.007**	C/T	---	---
Beaver	0.000	C	---	---	Gravel/Railroad	**0.008**	C/T	---	---
Beaver	0.001	C	---	---	Gravel/Railroad	**0.009**	C/T	---	---
Beaver	0.001	C	---	---	Gravel/Railroad	**0.012**	C/T	---	---
*Beaver*	0.003	C	---	---	Gravel/Railroad	**0.021**	C/T	---	---
*Beaver*	**0.006**	C/T	---	---	Gravel/Railroad	**0.032**	C/T	---	---
Bett’s	0.000	C	---	---	Gravel/Railroad	**0.041**	C/T	---	---
Bett’s	**0.007**	C/T	---	---	Gravel/Railroad	0.998	T	---	---
Bowl	1.000	T	0.000	C	Gravel/Railroad	0.999	T	---	---
Bowl	0.000	C	0.000	C	Gravel/Railroad	0.999	T	---	---
Bowl	1.000	T	0.000	C	Gravel/Railroad	1.000	T	---	---
Bowl	1.000	T	**0.845**	T/C	Gravel/Railroad	1.000	T	---	---
Bowl	1.000	T	**0.847**	T/C	Gravel/Railroad	1.000	T	---	---
Bowl	0.996	T	**0.858**	T/C	Moores	0.000	C	---	---
Bowl	1.000	T	**0.921**	T/C	Moores	0.000	C	---	---
Bowl	1.000	T	**0.926**	T/C	Moores	**0.008**	C/T	---	---
Bowl	1.000	T	**0.937**	T/C	New Street	0.002	C	---	---
Bowl	1.000	T	**0.952**	T/C	Ocean City	0.000	C	---	---
Bowl	1.000	T	**0.955**	T/C	Ocean City	0.000	C	---	---
Bowl	0.000	C	1.000	T	Ocean City	0.000	C	---	---
Bowl	0.004	C	1.000	T	Ocean City	0.000	C	---	---
*Bowl*	**0.028**	C/T	0.997	T	Ocean City	0.000	C	---	---
*Bowl*	**0.005**	C/T	0.998	T	Ocean City	0.000	C	---	---
Bowl	**0.016**	C/T	0.999	T	Ocean City	0.000	C	---	---
Bowl	0.000	C	0.999	T	Ocean City	**0.025**	C/T	---	---
Bowl	0.004	C	0.999	T	Ocean City	**0.040**	C/T	---	---
Bowl	0.000	C	---	---	Ocean City	**0.049**	C/T	---	---
Bowl	0.000	C	---	---	Polpis	0.001	C	---	---
Bowl	0.000	C	---	---	UT	**0.994**	T/C	0.000	C
Bowl	0.000	C	---	---	UT	1.000	T	0.000	C
Bowl	0.001	C	---	---	UT	1.000	T	0.000	C
Bowl	0.002	C	---	---	UT	1.000	T	0.000	C
Bowl	**0.006**	C/T	---	---	UT	1.000	T	0.001	C
Bowl	**0.007**	C/T	---	---	UT*	**0.994**	T/C	**0.853**	T/C
Bowl	**0.008**	C/T	---	---	UT	0.998	T	**0.877**	T/C
Bowl	**0.013**	C/T	---	---	UT	1.000	T	**0.984**	T/C
Bowl	**0.410**	C/T	---	---	UT	0.999	T	0.999	T
Bowl	1.000	T	---	---	UT	**0.983**	T/C	0.999	T
Bowl	1.000	T	---	---	UT	**0.993**	T/C	1.000	T
Bowl	1.000	T	---	---	UT	0.000	C	---	---
Cemetery	0.000	C	0.997	T	UT	0.000	C	---	---
Cemetery	0.000	C	---	---	UT	0.000	C	---	---
Cemetery	0.003	C	---	---	UT	0.000	C	---	---
Cemetery	**0.006**	C/T	---	---	UT	0.000	C	---	---
Cemetery	**0.012**	C/T	---	---	UT	0.001	C	---	---
Cemetery	**0.015**	C/T	---	---	UT	0.001	C	---	---
Cemetery	**0.320**	C/T	---	---	UT	0.002	C	---	---
Cemetery	**0.664**	T/C	---	---	UT	0.003	C	---	---
Cemetery	1.000	T	---	---	UT	0.003	C	---	---
Clift Rd	0.003	C	---	---	UT	**0.007**	C/T	---	---
Grand Gorge	1.000	T	---	---	UT	**0.009**	C/T	---	---
Grand Gorge	1.000	T	---	---	UT	**0.019**	C/T	---	---
*Gravel/Railroad*	1.000	T	0.000	C	UT	**0.028**	C/T	---	---
Gravel/Railroad	0.000	C	0.000	C	UT	**0.039**	C/T	---	---
*Gravel/Railroad*	0.999	T	0.000	C	UT	**0.045**	C/T	---	---
Gravel/Railroad	0.999	T	0.000	C	UT	0.996	T	---	---
Gravel/Railroad	0.996	T	0.000	C	UT	1.000	T	---	---
*Gravel/Railroad*	**0.027**	C/T	0.002	C	UT	1.000	T	---	---
Gravel/Railroad	0.000	C	**0.925**	T/C	UT	1.000	T	---	---

Heteroplasmic inds. bolded; doubly heteroplasmic inds. indicated by ‘*’. Italics denote inds. verified by Sanger sequencing.

### The magnitude of linkage disequilibrium

In order to assess the magnitude of linkage disequilibrium between the SNPs assayed in the *Cox1* and *Atp9* genes, we calculated linkage disequilibrium (as *D*) following the methods of McCauley & Ellis [7] and McCauley [2]. We used the equation from [16], *D* = *x*_*ij*_ − *p*_*i*_ *q*_*j*_, where *x*_*ij*_ is the observed frequency of individuals with a particular two-locus genotype, *p*_*i*_ is the frequency of the relevant allele at the first locus (*Cox1* SNP: T or C), and *q*_*j*_ is the frequency of the relevant allele at the second locus (*Atp9* SNP: T or C). We standardized this value by dividing *D* by *D*_*max*_, the maximum value of *D* possible given the observed allele frequencies *p*_*i*_, (1 − *p*_*i*_), and *q*_*j*_, (1 − *q*_*j*_) [16], to obtain *D’*. By doing this, standardized linkage disequilibrium *D’* then ranges from −1 to 1 with *D’* = 0 indicating linkage equilibrium. The expected frequencies for the two-locus genotypes were calculated from the products of allele frequencies at the two loci. Finally, a statistical association between the SNP at *Cox1* and the SNP at *Atp9* (i.e., whether the variant at one gene was independent of the variant at the other gene) was tested using a Fisher’s Exact Test [17].

## Results

For both assays, the Ct values obtained from replicates/individual were quite repeatable (Spearman’s Rank Correlation of the paired Ct values r_s_ = 0.924 for the *Cox1* assay and r_s_ = 0.893 for the *Atp9* assay; p < 0.001 in both cases with ‘U’ results assigned an arbitrary Ct numeric score of 60 as outlined above) (Tables B and C in S1 File). Each individual was genotyped twice for both mitochondrial genes, and these Ct values resulted in two quantifications of heteroplasmy in those genes for the individuals meeting the above criteria for inclusion in the data set. For each individual/gene, these two scores were averaged. In previous works, a minimum threshold frequency of 0.005 (0.5%) of the minority SNP variant was used to define an individual as heteroplasmic [8,9]. Here we also use an average minority frequency of 0.005 or greater as necessary to define an individual as heteroplasmic for that SNP. Note that in all cases, the majority SNP that was obtained from the Q-PCR assay matched that of the Sanger sequencing results. In three individuals from the *Atp9* assay, the Q-PCR reaction failed i.e., showed no signal for T or C, and these individuals were indeed the third variant (the ‘A’) when sequenced (data not shown).

For the *Cox1* SNP marker 43 of 140 individuals (30.7%) were heteroplasmic with each of the two *Cox1* SNP variants serving as the minority type in at least some heteroplasmic individuals (Tables 2 and 3; Table D in S1 File). While the majority-minority distribution was quite uneven in most heteroplasmic individuals, the most even mix of variants was 0.590:0.410, as observed in one individual. Of the remaining 97 individuals, 57 (58.8%) were homoplasmic for the ‘C’ variant and 40 (41.2%) homoplasmic for the ‘T’ variant at the SNP site of interest. For the *Atp9* SNP marker, 15 of 50 individuals (30.0%) were heteroplasmic (Tables 2 and 3; Table E in S1 File). Here only the ‘C’ *Atp9* variant was observed as the minority type in heteroplasmic individuals—an asymmetry also observed in cultivated carrot where the ‘T’ variant was never observed as a minority haplotype [15]. Of the remaining 35 individuals, 14 (40.0%) were homoplasmic for the ‘C’ variant and 21 (60.0%) were homoplasmic for the ‘T’ variant at the SNP site of interest. Again, the majority-minority distribution was quite uneven in most heteroplasmic individuals, with 0.845:0.155 being the most even mixture observed.

**Table 3:** Summary genotype counts for *Cox1* and *Atp9* as given by either homoplasmic T or C and heteroplasmic T/C or C/T where the major SNP is listed first.

*Cox1*	No. Individuals	*Atp9*	No. Individuals
T	40	T	21
C	57	C	14
T/C	5	T/C	15
C/T	38	C/T	0
Total	140	Total	50

Heteroplasmy was observed in both the *Cox1* and the *Atp9* markers in two individuals. Considering both markers, heteroplasmy was observed in individuals found in eight of the 13 populations sampled. These included populations located in all four U.S. states in which the sampled natural populations were located (Table 1). When the two-locus genotypes are considered for the 50 individuals (Table 2) for which both *Cox1* and *Atp9* genotypes were obtained (with the majority variant considered the SNP genotype in the members of that group that were heteroplasmic) individuals carrying all four possible *Cox1*/*Atp9* combinations of nucleotides were observed. These included 15 T/T, 11 T/C, 21 C/T, 3 C/C individuals (Table 4). Applying the methods of McCauley & Ellis [7] and McCauley [2] for calculating linkage disequilibrium and the equations from [16] to the 50 two-locus SNP genotypes described above, we found that **|** *D*’ = 0.511**|**. It should be noted that given the relatively small sample size used here to calculate LD, this value should be viewed as only an estimate of the degree of LD between these two genes. Still, the Fisher’s Exact Test indicated that the variants at the two loci were not randomly associated (df = 1, p < 0.05).

**Table 4:** Observation of the four-gamete rule and observed and expected two-locus nucleotide combinations.

*Cox1* SNP	*Atp9* SNP	No. Ind. Obs.	No. Ind. Exp.
T	T	15	19
T	C	11	7
C	T	21	17
C	C	3	7

No. Ind. Obs. = number of individuals observed and No. Ind. Exp. = number of individuals expected when D’ = 0.

## Discussion

Here we discovered somewhat surprising levels of sequence heteroplasmy in natural populations of wild carrot. We previously documented some of the genotypic consequences of recombination in the mitochondrial genome of *D*. *carota* and speculated on the occurrence of mitochondrial heteroplasmy [13]. The current results suggest that at least low-level heteroplasmy of the mitochondrial genome, defined as such, is quite common among individuals found over much of the distribution of wild carrot in the eastern U.S., if not more widely. Few studies have characterized heteroplasmy in natural populations, but work in wild collected individuals of *S*. *vulgaris* found that heteroplasmy was moderately common with roughly 15% of individuals considered as heteroplasmic [8]. It should also be noted that heteroplasmy is necessarily defined here, and as in the study by Pearl et al. [8], by the collection of mitochondrial genomes found within a 2 cm square portion of leaf tissue, rather than at the level of the whole plant or individual cell.

Considering cases where paternal leakage leads to sequence heteroplasmy, previous studies of *S*. *vulgaris* demonstrate that heteroplasmy can be transmitted across generations via mostly maternal inheritance of mitochondria, or lost from maternal lines owing to random processes during cell division resulting in homoplasmy in that cell line (vegetative segregation) [8,9]. In order for paternal leakage to result in detectable heteroplasmy in open-pollinated natural populations, such as studied here, the pollen donor and recipient must differ with regard to marker genotype and so heteroplasmy should be difficult to detect in those local populations displaying low levels of marker polymorphism. In our previous work, we found a moderate number of wild carrot populations harboring mitochondrial DNA polymorphism [13], still many populations studied previously, and here, have little to no variation within the genes used as markers. This factor, along with the detectability issue mentioned above, suggests our current study has likely underestimated heteroplasmy in natural populations of wild carrot.

The possibility of occasional transmission of the mitochondrial genome via paternal leakage resulting in heteroplasmy in wild carrot is particularly interesting if this predicts that such leakage could also be a mechanism of genetic exchange between wild and domesticated carrot at localities were the crop and weed grow in close proximity. Wild carrot and domesticated carrot are known to hybridize easily [10], but strict maternal inheritance would limit the exchange of the mitochondrial (and chloroplast) genome between the two entities [18,19]. Maternal inheritance could limit the potential for genetic modifications of the crop (targeted to an organellar genome) to be incorporated into the genome of the weed. Moreover, the likelihood of such leakage may also be enhanced in crop species that utilize CMS for hybrid seed production given that paternal transmission may be enhanced in species that exhibit gynodioecy and CMS [3]. Paternal leakage between crop and weed would facilitate the transfer of such modifications. We have begun further studies involving formal crosses between *D*. *carota* individuals of known marker genotypes in order to evaluate this scenario. An additional point to consider is that, as noted above, we observed an asymmetry in *Atp9* heteroplasmy in which the ‘T’ SNP was never found as the minority, and this bias was also seen in a recent crop carrot study [15]. If paternal leakage were the sole contributor to heteroplasmy, one might expect no bias in minority/majority mixture. This finding suggests that multiple factors may act in the generation and maintenance of heteroplasmy in carrot and certainly warrants further investigation.

The occurrence of heteroplasmy within an individual has important evolutionary implications as well, since within-individual genetic variation would increase the probability that the recombining molecules would be genetically differentiated. Thus heteroplasmy may be an important factor influencing the impact of inter-molecular recombination on plant mitochondrial genome evolution [1,2]. In plant mitochondrial genomes, intra- or inter-molecular recombination associated with repeat sequences, results in frequent structural rearrangements [20]. Yet, it is not known what level of heteroplasmy (evenness of majority/minority representation) is needed for biological significance in this regard. Evidence for the product of such recombination can be seen in the data set presented here. Our observation of the four possible two-locus nucleotide combinations (Table 4) is in keeping with the four-gamete rule suggested by Hudson & Kaplan [21] as a simple indicator of recombination between the nucleotides in question. This is because, barring recurrent mutation, recombination between two sequences would be required to generate the fourth combination in a sample containing three of the four possible two-locus combinations, including individuals polymorphic for at least two of the three combinations, as illustrated by Fig 1 in McCauley [2]. Finally, McCauley & Ellis [7] and McCauley [2] suggest that evidence of the consequences of recombination is also found in the magnitude of LD between the two variable sites in question. Again barring recombination, the standardized LD (*D’*) should have an absolute value of one, and **|** *D’* **|** would approach zero (linkage equilibrium) in populations with frequent recombination between the sites in question [16]. The finding here that **|** *D’* = 0.511**|** suggests substantial, but not rampant, recombination between *Cox1*/*Atp9* mitochondrial genotypes in *D*. *carota* and demonstrates the potential for sequence heteroplasmy to generate mitochondrial genotypic novelty in natural plant populations.

## Supporting Information

S1 File Supporting information. Additional supporting information may be found in the online version of this article and includes six tables described here. Assay Design Details (Table A). Mixture experiment data for *Cox1* assay (Table B). Mixture experiment data for *Atp9* assay (Table C). Raw data, analysis calculations, and quantitative heteroplasmy scores for 140 individuals assayed for *Cox1* (Table D). Raw data, analysis calculations, and quantitative heteroplasmy scores for 50 individuals assayed for *Atp9* (Table E).
(XLSX)
